# *microRNA-301b-3p* downregulation underlies a novel inhibitory role of long non-coding RNA MBNL1-AS1 in non-small cell lung cancer

**DOI:** 10.1186/s13287-019-1235-8

**Published:** 2019-05-21

**Authors:** Peng Li, Wenqun Xing, Jinliang Xu, Dongfeng Yuan, Guanghui Liang, Baoxing Liu, Haibo Ma

**Affiliations:** Department of Thoracic Surgery, The Affiliated Cancer Hospital of Zhengzhou University, Henan Cancer Hospital, No. 127, Dongming Road, Jinshui District, Zhengzhou, 450008 Henan Province People’s Republic of China

**Keywords:** Non-small cell lung cancer, Long non-coding RNA MBNL1-AS1, * microRNA-301b-3p*, TGFBR2, Drug resistance, Proliferation, Invasion

## Abstract

**Background:**

Non-small cell lung cancer (NSCLC) is the second most prevalent cause of cancer-related fatality. Long non-coding RNAs (lncRNAs) have been observed to exercise functions in NSCLC. Here, the current study aimed to explore the potential mechanism of lncRNA MBNL1-AS1 in NSCLC.

**Methods:**

Microarray analysis was performed to screen the differentially expressed lncRNA associated with NSCLC and its potential mechanism. The lncRNA MBNL1-AS1 expression was quantified in 56 paired NSCLC and adjacent normal tissue samples. In an attempt to outline the function of lncRNA MBNL1-AS1 in NSCLC and to identify the interaction among lncRNA MBNL1-AS1, *microRNA-301b-3p* (miR-301b-3p) and TGFBR2, ectopic expression, depletion, and reporter assay experiments were conducted to detect CSC proliferation, migration, invasion, drug resistance, and sphere formation in NSCLC.

**Results:**

Initially, the intersection among lncRNA MBNL1-AS1, miR-301b-3p, and TGFBR2 was observed in NSCLC. While a poor expression of lncRNA MBNL1-AS1 and TGFBR2, along with a high expression of miR-301b-3p was observed in NSCLC tissues. A demonstration of lncRNA MBNL1-AS1 restoration significantly decreased CSC proliferation, migration, invasion, drug resistance, and sphere formation in NSCLC. LncRNA MBNL1-AS1 functioned as a sponge of miR-301b-3p, which inverted the inhibitory role of lncRNA MBNL1-AS1 in CSC proliferation, migration, invasion, drug resistance, and sphere formation in NSCLC. LncRNA MBNL1-AS1 positively regulated TGFBR2 which was a target gene of miR-301b-3p. At last, upregulated lncRNA MBNL1-AS1 or depleted miR-301b-3p suppressed the xenograft tumor formation in vivo.

**Conclusion:**

Collectively, the present study suggests an inhibitory role of lncRNA MBNL1-AS1 in CSC drug resistance of NSCLC by upregulating miR-301b-3p-targeted TGFBR2.

## Introduction

Non-small cell lung cancer (NSCLC) has been a persistent cause of cancer-related mortality worldwide, accompanied by a comparatively low 5-year survival rate [1]. NSCLC metastatic progression is correlated with the high mortality rates associated with NSCLC [2]. Early diagnosis of NSCLC has become a clinical essential in planning and providing effective treatment, which markedly improves the overall survival rate of patients with NSCLC [3]. However, the prognosis of patients suffering from NSCLC remains to be unfortunate [4]. In recent years, the theory of cancer stem cells (CSCs), that cancers are maintained by subpopulations of tumor cells with features of stem cells and progenitors, has served as a focus of in-depth investigations in cancer research [5]. A research reported the principal involvement of CSCs in tumor progression, recurrence, and drug resistance [6]. CSCs due to their high tumorigenicity can be observed in multiple human cancers, with great significance in NSCLC [7]. Targeting CSCs has been therapeutically proven as a curative strategy for NSCLC treatment [8], while the specific mechanisms still remain as a topic of research. Thus, it is necessary to develop new treatment strategies against CSCs as a mode of better clinical intervention in NSCLC.

Long non-coding RNAs (lncRNAs), comprising of over 200 nucleotides in length, with vital functionality as precarious modulators of cancer biology with significant impacts on cell proliferation, metastasis, and apoptosis [9]. MBNL has been proven as a participant in the modulation of selective splicing of pre-mRNA [10]. MBNL1 plays a vital role in the initiation of colorectal cancer (CRC) by reducing the expression of microRNAs (miRNAs/miRs) [11]. LncRNA MBNL1-AS1 has been elucidated to be downregulated in NSCLC [12]. Existing literature has implicated the functionality of various miRNAs like miR-504 as facilitators along the progression of NSCLC [13], thereby shedding light on the urgency for identification of novel valuable therapeutic targets for NSCLC. A clinically proven correlation has been implicated by miR-301b with vasoconstriction in pulmonary hypertension by regulating a target gene [14]. The participation of miR-301b-3p was proven to be vital in myogenic differentiation by targeting Rb1-inducible coiled-coil 1 [15]. Wu et al. have confirmed the presence of an aberrantly expressed miR-301b along the progression of lung cancer [16]. Furthermore, microarray analysis from the current study revealed and verified the transforming growth factor beta receptor II (TGFBR2) as a target of miR-301b-3p. The TGFBR2 belongs to the serine/threonine protein kinase family and the TGF-β receptor subfamily, and cases of disrupted TGF-β pathway have been demonstrated to stimulate tumor progression [17]. Accumulating evidence from a previous study revealed that TGFBR2 depletion also implicates along the progression of carcinogenesis of NSCLC [18]. With the abovementioned literature serving as a hypothesis, we obtained NSCLC tissues and adjacent normal tissues to explore and develop tumorigenicity assay in nude mice in order to verify the conceivable effects of lncRNA MBNL1-AS1 on the cellular processes of NSCLC CSCs via miR-301b-3p and TGFBR2.

## Materials and methods

### Ethics statement

The study was performed with the approval of the Ethics Committee of The Affiliated Cancer Hospital of Zhengzhou University, Henan Cancer Hospital. All patients signed informed consents prior to tissue collection, which was in compliance with the Declaration of Helsinki [19]. Moreover, the animal experiment procedures were performed in accordance with the protocols issued by the Institutional Animal Research Committee of The Affiliated Cancer Hospital of Zhengzhou University, Henan Cancer Hospital. Measures were taken to minimize animal suffering.

### Microarray analysis

Chip data relative to NSCLC, including GSE101929, GSE102286, and GSE33532, were retrieved from the Gene Expression Omnibus (GEO) database (https://www.ncbi.nlm.nih.gov/geo/), from which GSE102286 emerged as the miRNA expression chip. The Affy package of R language (http://www.bioconductor.org/packages/release/bioc/html/affy.html) was employed for standard pretreatment of the chip data, and the limma package of R language (http://master.bioconductor.org/packages/release/bioc/html/limma.html) was employed so as to identify the differentially expressed genes (DEGs). The heat maps were drawn using the pheatmap package (https://cran.r-project.org/web/packages/pheatmap/index.html). The subcellular localization of lncRNA with the differential expression in GSE101929 was further analyzed using the LncATLAS database (http://lncatlas.crg.eu/) in an attempt to speculate the potential regulatory mechanism of lncRNA. The RAID database (http://www.rna-society.org/raid/index.html) was employed to predict the miRNA targeted by lncRNA, and jvenn (http://jvenn.toulouse.inra.fr/app/example.html) was used for a comparative analysis of the predicted miRNA results and the differential miRNA in GSE102286 as per to screen the miRNA. The regulatory target genes of miRNA were predicted in the mirDIP (http://ophid.utoronto.ca/mirDIP/), miRDB (http://www.mirdb.org/), miRSearch (http://www.exiqon.com/microrna-target-prediction), RNA22 (https://cm.jefferson.edu/rna22/), and miRTarBase databases (http://mirtarbase.mbc.nctu.edu.tw/php/search.php), and the predicted results were compared with the DEGs in GSE33532 in order to sequentially screen out the respective DEGs.

### Specimen collection

The NSCLC tissues and adjacent normal tissues samples (at least 5 cm from the edge of NSCLC mass) were collected from 56 patients (Table 1) with NSCLC who underwent surgical intervention in The Affiliated Cancer Hospital of Zhengzhou University, Henan Cancer Hospital from 2016 to 2017. None of the patients were administered chemotherapy or radiotherapy prior to the study.

**Table 1:** Correlations between LncRNA MBNL1-AS1 and clinicopathological features of NSCLC patients (*n* = 56)

Variables		lncRNA MBNL1-AS1		
	*n*	High	Low	*p* value
Age				
< 50	33	19 (57.58)	14 (42.42)	0.5882
≥ 50	23	11 (47.83)	12 (52.17)	
Gender				
Male	35	22 (62.86)	13 (37.14)	0.0990
Female	21	8 (38.10)	13 (61.90)	
Pathology				
Squamous carcinoma	20	9 (45.00)	11 (55.00)	0.2312
Adenocarcinoma	32	20 (62.50)	12 (37.50)	
Large cell carcinoma	4	1 (25.00)	3(75.00)	
T classification				
T1 + T2	40	24 (60.00)	16 (40.00)	0.1494
T3 + T4	16	6 (37.50)	10 (62.50)	
*N* classification				
N0	17	13 (76.47)	4 (23.53)	0.0401
N1 + N2	39	17 (43.59)	22 (56.41)	

*Note: lncRNA* long non-coding RNA, *MBNL1* muscleblind-like 1, *NSCLC* non-small cell lung cancer

### Cell culture

The human normal lung epithelial cell line BEAS-2B and NSCLC cell lines (A549, H1299, and SK-MES-1) were purchased from the American Type Culture Collection (ATCC, Manassas, VA, USA). The aforementioned cells were inoculated in the RPMI-1640 medium (11875093, Invitrogen, Carlsbad, CA, USA) containing 10% fetal bovine serum (FBS, 10100147, Invitrogen, Carlsbad, CA, USA), respectively, then combined with 100 g/mL penicillin/streptomycin (15140122, Invitrogen, Carlsbad, CA, USA) and cultured in a 5% CO_2_ incubator at 37 °C.

### Flow cytometry

According to a previous study [20], the NSCLC cells were stained with 5 μg/mL Hoechst 33342 (B2261, Sigma, St. Louis, MO, USA) irrespective of the presence or absence of 50 μM verapamil (V105, Sigma-Aldrich Chemical Company, St Louis, MO, USA), followed by staining with 2 μg/mL propidium iodide (PI, P4170, Sigma-Aldrich Chemical Company, St Louis, MO, USA). The cells were sorted in a FACS-Aria III flow cytometer (BD Biosciences, San Jose, CA, USA). The low Hoechst Red and low Hoechst Blue and the missing area of the verapamil group were set and prepared for the supplementary population (gate of SP cells). The cells were initially gated so as to sort the SP^+^ cells and then analyzed for CD133 expression.

### RNA isolation and quantitation

Total RNA was extracted using the Trizol Kit (10296010, Invitrogen, Carlsbad, CA, USA). The primer sequences were synthetized by BGI Co., Ltd. (Shenzhen, China) (Table 2). Next, for RT-qPCR of miRNAs, 100 ng of total RNA was reverse-transcribed and subjected to Taqman® miRNA assay (Applied Biosystems), and for RT-qPCR of mRNAs, cDNA synthesis was performed with 1 μg of total RNA according to the instructions of the EasyScript First-Strand cDNA Synthesis SuperMix (AE301-02, Beijing TransGen Biotech Co., Ltd., Beijing, China). The ABI7500 quantitative PCR instrument was employed for RT-qPCR following the instructions of the SYBR®Premix Ex TaqTM II kit. The level of miR-301b-3p was normalized to the housekeeping gene U6, while the expression level of lncRNA MBNL1-AS1 and TGFBR2 was normalized to the housekeeping gene glyceraldehyde-3-phosphate dehydrogenase (GAPDH). The expression ratio of the target gene between the experimental and control groups was calculated using the 2^−ΔΔCt^ method. The experiment was conducted three times.

**Table 2:** Primer sequences for RT-qPCR

Genes	Primer sequences (5′-3′)
miR-301b-3p	F: CAGGTGCTCTGACGAGGTTG
	R: TGGTCCCAGATGCTTTGACA
U6	F: CTCGCTTCGGCAGCACATA
	R: AACGATTCACGAATTTGCGT
lncRNA MBNL1-AS1	F: CTCCCGCTTCTTCTACCGAC
	R: TTGGTGCATTTTAAGGCGGC
TGFBR2	F: GTAGCTCTGATGAGTGCAATGAC
	R: CAGATATGGCAACTCCCAGTG
GAPDH	F: GAAGGTGAAGGTCGGAGTC
	R: GAAGATGGTGATGGGATTTC

*Note: RT-qPCR* reverse transcription quantitative polymerase chain reaction, *MBNL1-AS1* muscleblind-like 1-antisense RNA 1, *miR-301b-3p*, *microRNA-301b-3p*, *GAPDH* glyceraldehyde-3-phosphate dehydrogenase, *lncRNA* long non-coding RNA, *TGFBR2* transforming growth factor beta receptor II, *F* forward, *R* reverse

### G418 screening

The preliminary experiment was conducted to verify the extermination concentration of antibiotics (the minimum cell death concentration was regarded as the best screening concentration). After 24 h of transfection, G418 with the best screening concentration was added for the screening of stable transgenic plants. Briefly, upon termination of a large number of cells after 6 days of culturing, the cells were further incubated with increasing serum concentration. After 10 days of incubation, the concentration of G418 had reduced by half to maintain the optimum screening pressure. On the 14th day after the screening, the resistant clone occurred. After selection and large amplification of the monoclone using the limiting dilution assay, the total RNA was extracted in order to determine the expression of the target gene using RT-qPCR.

### Sphere formation

CSCs were cultured in serum-free Dulbecco’s modified Eagle medium (DMEM)/F12 medium (11320-033, Gibco, Carlsbad, CA, USA) supplemented with a combination of insulin (Sigma-Aldrich Chemical Company, St Louis, MO, USA), 20 ng/mL human recombinant epidermal growth factor (EGF, Peprotech, Rocky Hill, NJ, USA), and 10 ng/mL basic fibroblastic growth factor (bFGF, Peprotech, Rocky Hill, NJ, USA). Sphere formation was assessed 2 weeks after seeding, and the tumor sphere was observed under a microscope (Olympus, Tokyo, Japan).

### Fluorescence in situ hybridization (FISH)

The subcellular localization of lncRNA MBNL1-AS1 was detected using the FISH kit (BIS-P0001, Guangzhou Bersin Biotechnology Co., Ltd., Guangzhou, China). The NSCLC CSC slide was added with the lncRNA MBNL1-AS1 probe hybridization solution labeled by Digoxigenin, while the antagonistic lncRNA MBNL1-AS1 probe served as a negative control (NC). The slide was hybridized at 42 °C for 16 h and immersed in 2 × SSC, followed by subsequent immersion in 70% ethanol for 3 min and staining with 4′6-diamidino-2-phenylindole (DAPI) for 5–10 min. The slide was photographed under a confocal laser-scanning microscope to document the observations. The experiment was conducted three times. All images were obtained under the Zeiss LSM880 NLO (2 + 1 with BIG) confocal microscope (Leica Microsystems, Mannheim, Germany).

### Cell counting kit-8 (CCK-8)

Cells were seeded into 96-well plates at a density of 2 × 10^3^ cells/well and incubated with 5% CO_2_ at 37 °C for cell adherence. Each well was added with 10 uL of the CCK-8 reagent, and then, the optical density (OD) value was measured at a wavelength of 450 nm at variable time points of 24, 48, 72, and 96 h.

Then, the cell resistance was evaluated following procedures from existing research [21]. A total of 5 × 10^3^ cells were seeded into the 96-well plates and incubated for 24 h after transfection. Next, the cells were further incubated in variable concentrations of gefitinib (SML1657, Sigma-Aldrich Chemical Company, St Louis, MO, USA) or cisplatin (P4394, dissolved in 0.15 M NaCl, Sigma-Aldrich Chemical Company, St Louis, MO, USA) for 48 h. The cells were incubated with the CCK-8 reagent at 37 °C for 4 h. The OD value was measured at a wavelength of 570 nm using a microplate reader (Sunrise Microplate Reader, TECAN, Switzerland).

### Clone formation assay in vitro

Transfected cells were seeded into 6-well plates at a density of 400 cells/well and incubated in DMEM supplemented with 10% FBS and 5% CO_2_ at 37 °C for 2 weeks. The clone cells were fixed in 95% alcohol and stained with 0.1% crystal violet for 10 min. Finally, the total number of clones (a clone was regarded as > 50 cells) were counted.

### Transwell assay

For transwell assay, 24-well transwell plates (diameter of 8 μm, Corning Incorporated, Corning, NY, USA) were employed so as to measure the cell migratory and invasive ability. The 5 × 10^4^ cells were seeded into an apical chamber covered with 200 mg/mL of Matrigel (1:8, Yepsen Company, Shanghai, China). Then, the medium containing the serum was added to the basolateral chamber. After incubation for 24 h, the cells invading into the basolateral chamber through Matrigel were fixed in 100% methanol for 10 min, stained with 0.1% crystal violet for 10 min, and then observed and counted under a microscope (× 200, Leica Inc., Wetzlar, Germany) with 5 random fields selected.

### Scratch test

NSCLC CSCs were seeded into 6-well plates at a density of 2.5 × 10^4^ cells/cm^2^, and the 10 μL scratch was prepared. Subsequently, the samples were washed with phosphate-buffered saline (PBS) two times and incubated in the DMEM containing 10% FBS in a 5% CO_2_ incubator with saturated humidity at 37 °C. Images were obtained at 0 h and 24 h under an inverted microscope and analyzed using ImageJ software. The distance between the cells on both sides of the scratch at each time point (μm) was recorded. The migration distance of cells was calculated as an equivalent obtained by subtracting the distance between the scratch edge at 0 h from the migration edge at 24 h, which indicated the migratory ability of cells. Three replicates were set for each group.

### Western blot analysis

Western blot analysis was conducted as previously reported [22]. In brief, the total protein was extracted from tissues and cells using radio-immunoprecipitation assay (RIPA) cell lysis supplemented with phenylmethyl sulfonylfluoride (PMSF) (R0010, Beijing Solarbio Science & Technology Co. Ltd., Beijing, China). The extracted proteins were separated by performing sodium dodecyl sulfate-polyacrylamide gel electrophoresis (SDS-PAGE) and then transferred onto a polyvinylidene fluoride (PVDF) membrane (FFP36, Beyotime Institute of Biotechnology, Shanghai, China). The membrane was blocked with 5% bovine serum albumin (BSA) at 37 °C for 2 h and incubated overnight at 4 °C with the primary antibodies against rat anti-GAPDH monoclonal antibody (A21994, 0.125 μg/mL, Invitrogen, Carlsbad, CA, USA), rabbit anti-sekclsky mothers against dpp (SMAD)2/SMAD3 polyclonal antibody (SAB2702053/SAB2702052, 1: 1000), rabbit anti-phospho-SMAD2/SMAD3 polyclonal antibody (SAB4300252/SAB4504210, 1: 1000), rat anti-Oct4 monoclonal antibody (P0082, 1 μg/mL), rat anti-ATP-binding cassette superfamily G (White) member 2 (ABCG2) monoclonal antibody (SAB1403023, 5 μg/mL), and rabbit anti-TGFBR2 polyclonal antibody (AV44743). The abovementioned antibodies were acquired from the Sigma-Aldrich Chemical Company (St Louis, MO, USA) except the rat anti-GAPDH monoclonal antibody. Subsequently, the membrane was incubated with the goat ant-mouse immunoglobulin G (IgG) (ab205719, 1: 20000, Abcam, Cambridge, MA, USA) and goat anti-rabbit IgG (ab6721, 1: 20000, Abcam, Cambridge, MA, USA) at room temperature for 1 h. GAPDH served as the internal reference.

### RNA pull-down assay

The Magnetic RNA-Protein Pull-Down kit (20164, Pierce, Rockford, IL, USA) was employed so as to detect the binding situation between lncRNA MBNL1-AS1 and miR-301b-3p, and miR-301b-3p and TGFBR2. The lysate supernatant of NSCLC CSCs was divided into several parts (one part served as the input). According to the instructions, the cell lysate was incubated with the biotin-labeled miR-301b-3p, miR-NC, lncRNA MBNL1-AS1-wild type (wt), lncRNA MBNL1-AS1-mutant (mut), TGFBR2-wt and TGFBR2-mut, and streptavidin-labeled magnetic beads. Finally, the binding miRNA was analyzed by RT-qPCR.

### Dual-luciferase reporter gene assay

The binding sites between miR-301b-3p and lncRNA MBNL1-AS1, miR-301b-3p, and TGFBR2 were identified using the biological prediction website (https://cm.jefferson.edu/rna22/Interactive/). The dual-luciferase reporter gene essay was performed to identify the target relationship between miR-301b-3p and lncRNA MBNL1-AS1, and miR-301b-3p and TGFBR2. According to the verified binding sites, the wt of lncRNA MBNL1-AS1 and 3′-untranslated region (3′-UTR) of TGFBR2 mRNA were artificially synthesized, after which the mutant site of the complementary sequence in the seed sequence was designed in the wt of lncRNA MBNL1-AS1 and TGFBR2, which was inserted into the pGL3-basic (P2129, Shanghai Hewu Biotechnology Co., Ltd., Shanghai, China) plasmids. Following the manufacturer’s instructions of the Lipofectamine 2000 regents (Invitrogen, Carlsbad, CA, USA), miR-301b-3p mimic and inhibitor were respectively co-transfected with pGL3-lncRNA MBNL1-AS1-wt, pGL3-lncRNA MBNL1-AS1-mut, pGL3-TGFBR2-wt 3′-UTR, and pGL3-TGFBR2-mut 3′-UTR into NSCLC CSCs. The luciferase activity was measured in a GLoma × 20/20 Luminometer from Promega (E5311, Shaanxi Zhongmei Biotechnology, Co., ltd., Shaanxi, China) using the Dual-Luciferase Reporter Assay System (E1910, Promega, Madison, WI, USA). The experiment was conducted three times in order to obtain mean values.

### Tumorigenicity assay in nude mice

About 1 × 10^7^ cells were subcutaneously injected into the armpits of 5 specific-pathogen-free female BALB/c nude mice (aging 4–6 weeks and weighing 18–22 g, Hunan SJA Laboratory Animal Co., Ltd., Changsha, China) without thymus. During the 4th week after injection, the nude mice were euthanatized and the tumor was resected and fixed in 4% paraformaldehyde.

### Statistical analysis

Statistical analysis was performed using the SPSS 21.0 software (IBM Corp. Armonk, NY, USA), and the measurement data were expressed as mean ± standard deviation (SD). All experiments were conducted three times to obtain mean values. Comparisons between NSCLC and adjacent normal tissues were assessed by the paired *t* test, and comparisons between two groups were analyzed using the unpaired *t* test. Comparisons among multiple groups were highlighted by one-way analysis of variance (ANOVA), and pairwise comparisons of mean values among multiple groups were analyzed using the Tukey post hoc test. Correlation of lncRNA MBNL1-AS1 with a prognosis of patients with NSCLC was analyzed by Kaplan-Meier curve, and the data at different time points were analyzed by repeated measurement ANOVA. A value of *p* < 0.05 was considered to be statistically significant.

## Results

### LncRNA MBNL1-AS1 may affect NSCLC through miR-301b-3p-targeted TGFBR2

**Fig. 1:**
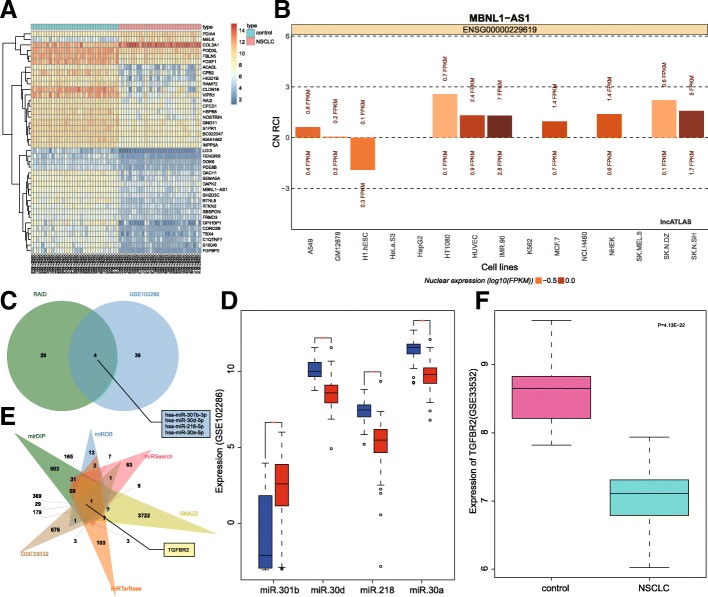
LncRNA MBNL1-AS1 may affect NSCLC via TGFBR2 by sponging miR-301b-3p. **a** Heat map of the 50 DEGs retrieved from GSE101929. The abscissa indicated the sample number and the ordinate indicated the DEG. The upper right histogram indicated color gradation; each rectangle in the figure corresponded to the level of a gene in a sample. **b** Subcellular localization of lncRNA MBNL1-AS1 in LncATLAS. CN RCI refers to the ratio of lncRNA expression in cytoplasm and nucleus. FPKM refers to fragments per kilobase of exon model per million mapped fragments. **c** Comparisons of lncRNA MBNL1-AS1-targeted miRNAs in RAID database and differential miRNAs in GSE102286. **d** Expression of miR-301b, miR-30d, miR-218, and miR-30a in GSE102286 chip. Blue indicated the adjacent normal tissues and red indicated NSCLC tissues. **e** Comparisons of miR-301b-3p-targeted genes in mirDIP, miRDB, miRSearch, RNA22, and miRTarBase databases and DEGs from GSE33532 chips. **f** TGFBR2 expression in GSE33532 chips. DEGs, differentially expressed genes; MBNL1-AS1, muscleblind-like 1-antisense RNA 1; NSCLC, non-small cell lung cancer; TGFBR2, transforming growth factor beta receptor II

The vital step in calculating our experimental results, the objective of understanding whether lncRNA MBNL1-AS1 may, or may not, affect NSCLC through regulating the miR-301b-3p-targeted TGFBR2, began with screening out of respective DEGs the NSCLC-related chip dataset GSE101929, which comprised of the lncRNA expression data, based on the GEO database. Subsiding the DEG screening, the heat maps of the 50 DEGs were drawn (Fig. 1a), which were representative of a significantly downregulated lncRNA MBNL1-AS1 in NSCLC tissues. Moreover, lncRNA MBNL1-AS1 expression was higher in the cytoplasm with respect to LncATLAS (Fig. 1b). LncRNA MBNL1-AS1 was speculated to affect the NSCLC disease by absorbing miRNA. Thus, we predicted the miRNAs that were regulated by lncRNA MBNL1-AS1 in the RAID database, which highlighted 33 miRNAs. On comparing various parameters among the 33 miRNAs and differential miRNAs in GSE102286, 4 crucial intersected miRNAs were chosen for subsequent experimentation (Fig. 1c), including hsa-miR-301b-3p, hsa-miR-30d-5p, hsa-miR-218-5, and hsa-miR-30a-5p. The expression profiles of the 4 miRNAs in GSE102286 are presented in Fig. 1d, which revealed that miR-301b was upregulated, whereas the other 3 miRNAs exhibited a contradictory trend, while lncRNA MBNL1-AS1 was aberrantly downregulated (Fig. 1a). Therefore, our team of researchers speculated the functionality of lncRNA MBNL1-AS1 as a molecular sponge of miR-301b-3p to influence NSCLC. Then, the target genes of miR-301b-3p were predicted in the mirDIP, miRDB, miRSearch, RNA22, and miRTarBase databases. After comparing the results with DEGs in GSE33532 in order to obtain the intersected gene, TGFBR2 was selected (Fig. 1e). TGFBR2 was downregulated in NSCLC based on GSE33532 (Fig. 1f). Overall, these results served as evidence supporting that lncRNA MBNL1-AS1 absorbing miR-301b-3p could potentially affect NSCLC by targeting TGFBR2.

### LncRNA MBNL1-AS1 is downregulated in NSCLC tissues and cells

**Fig. 2:**
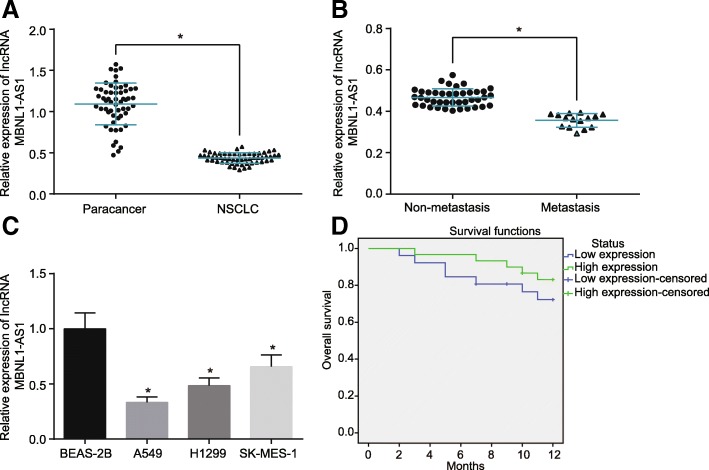
LncRNA MBNL1-AS1 is poorly expressed in NSCLC tissues and cells. **a** lncRNA MBNL1-AS1 expression in NSCLC tissues and adjacent normal tissues determined using RT-qPCR (*n* = 56). **b** lncRNA MBNL1-AS1 expression in tissues with and without lymph node metastasis determined using RT-qPCR. **c** lncRNA MBNL1-AS1 expression in NSCLC cell lines (A549, H1299, and SK-MES-1) and human normal lung epithelial cell line BEAS-2B determined using RT-qPCR. **d** Correlation of lncRNA MBNL1-AS1 with a prognosis of NSCLC patients analyzed by Kaplan-Meier curve; *p < 0.05 vs. the adjacent normal tissues, tissues without lymph node metastasis, or BEAS-2B cells. Measurement data were depicted as mean ± standard deviation; data in Fig. 2**a** was analyzed using a paired *t* test. Comparisons between two groups were analyzed using the unpaired *t* test, and comparisons among multiple groups were analyzed using one-way ANOVA with Tukey post hoc test used. The experiment was repeated three times. RT-qPCR, reverse transcription quantitative polymerase chain reaction; ANOVA, analysis of variance

Following our speculation if lncRNA MBNL1-AS1 could serve as a potential target to affect NSCLC by regulating miR-301b-3p and TGFBR2, with a supplementary aim to calculate the expression rate of lncRNA MBNL1-AS1 in NSCLC tissues; foremost, RT-qPCR was conducted in order to examine the expression of lncRNA MBNL1-AS1 in NSCLC tissues and adjacent normal tissues from 56 patients, tissues with and without lymph node metastasis, NSCLC cell lines (A549, H1299, and SK-MES-1), and human normal lung epithelial cell line BEAS-2B. The results showed that lncRNA MBNL1-AS1 expression in the NSCLC tissues and tissues with lymph node metastasis were lower than that observed in the adjacent normal tissues and tissues without lymph node metastasis, respectively (*p* < 0.05). The patients were divided based on the value among high expression (≥ 0.437) and low expression (< 0.437) (*p* < 0.05) with a mean value (0.437) in NSCLC tissues as the dividing line in between them (Fig. 2a and b). Meanwhile, lncRNA MBNL1-AS1 was downregulated in the A549, H1299, and SK-MES-1 cell lines compared to the BEAS-2B cell line (*p* < 0.05; Fig. 2c), with the A549 cell line exhibiting the lowest lncRNA MBNL1-AS1 expression. Thus, the A549 cell line was selected for further experimentation. Kaplan-Meier analysis revealed that the prognostic survival rate of NSCLC patients with a high expression of lncRNA MBNL1-AS1 was evidently higher than the prognostic survival rate observed due to a low expression of lncRNA MBNL1-AS1 (*p* < 0.05, Fig. 2d). These results demonstrated the downregulation of lncRNA MBNL1-AS1 in NSCLC tissues and cells.u

u

### Overexpressed lncRNA MBNL1-AS1 suppresses CSC proliferation, invasion, migration, drug resistance, and sphere formation in NSCLC

**Fig. 3:**
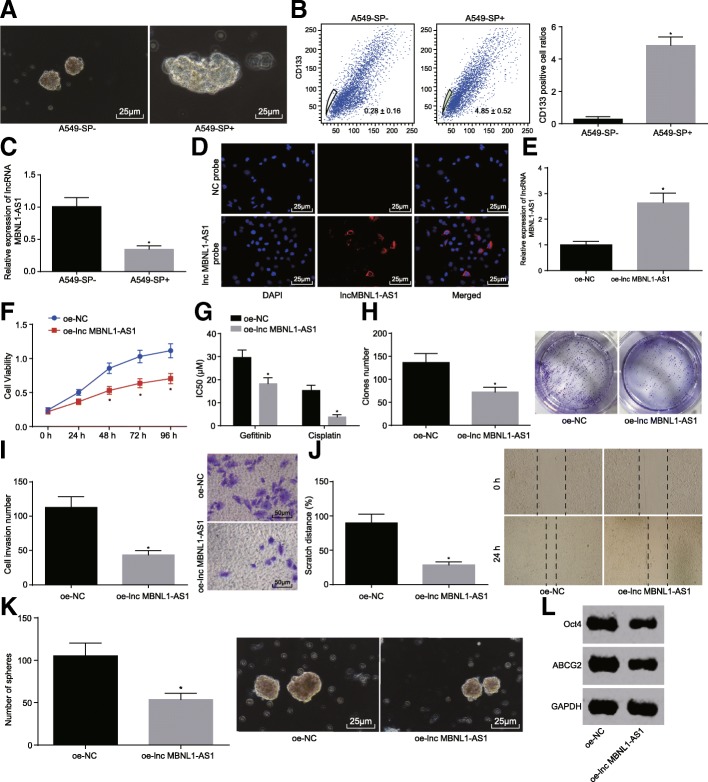
Elevated lncRNA MBNL1-AS1 represses A549-SP^+^ proliferation, migration, invasion, drug resistance, and sphere formation. **a** Sphere formation of A549-SP^+^ and A549-SP^−^ cells detected using sphere formation assay. **b** CD133-positive rate of A549-SP^+^ and A549-SP^−^ cells detected using flow cytometry. **c** lncRNA MBNL1-AS1 expression of A549-SP^+^ and A549-SP^−^ cells determined using RT-qPCR. **d** Localization of lncRNA MBNL1-AS1 in A549-SP^+^ verified using FISH. **e** lncRNA MBNL1-AS1 expression of A549-SP^+^ after lncRNA MBNL1-AS1 overexpression determined using RT-qPCR. **f** Proliferation of A549-SP^+^ after lncRNA MBNL1-AS1 overexpression detected using CCK-8. **g** Drug resistance of A549-SP^+^ against anti-tumor drugs after lncRNA MBNL1-AS1 overexpression detected using CCK-8. **h** Clone formation of A549-SP^+^after lncRNA MBNL1-AS1 overexpression detected using clone formation assay. **i** Cell invasion of A549-SP^+^ after lncRNA MBNL1-AS1 overexpression detected using transwell assay. **j** Cell migration of A549-SP^+^ after lncRNA MBNL1-AS1 overexpression detected using scratch test. **k** Sphere formation of A549-SP^+^ after lncRNA MBNL1-AS1 overexpression detected using sphere formation assay. **l** Protein levels of Oct4 and ABCG2 in A549-SP^+^ after lncRNA MBNL1-AS1 overexpression assessed using western blot analysis; *p < 0.05 vs. A549-SP^−^ or A549 cells treated with oe-lncRNA MBNL1-AS1. Measurement data were depicted as mean ± standard deviation. Comparisons between two groups were analyzed using unpaired *t* test. The experiment was repeated three times. Oct4, octamer 4; ABCG2, ATP-binding cassette superfamily G (White) member 2; CCK-8, cell counting kit-8; FISH, fluorescence in situ hybridization; NC, negative control

After determining the lncRNA MBNL1-AS1 expression in the NSCLC cells and tissues, flow cytometry was employed for stem cell sorting and identification in the A549 cell line in an attempt to sequentially examine the molecular mechanisms of lncRNA MBNL1-AS1 in CSCs of NSCLC. Sphere formation (Fig. 3a) and flow cytometry (Fig. 3b) presented with sophisticated sphere formation and CD133-positive rate of A549-SP^+^ cells compared to the A549-SP^−^ cells (*p* < 0.05), which in turn supported and aggravated the involvement and impact of A549-SP^+^ cells in NSCLC CSCs. Meanwhile, RT-qPCR showed that the lncRNA MBNL1-AS1 expression in CSCs of NSCLC was lower than the expression observed in non-CSCs (*p* < 0.05, Fig. 3c), and FISH supported the presence of lncRNA MBNL1-AS1 in the cytoplasm of NSCLC CSCs (*p* < 0.05, Fig. 3d). These findings established a correlation between the downregulation of lncRNA MBNL1-AS1 in NSCLC with the prognosis of the patients. In view of the low expression of lncRNA MBNL1-AS1 in NSCLC, we further studied its potential function upon lncRNA MBNL1-AS1 overexpression in CSCs of the NSCLC cell line A549. The cells were treated with an overexpression of lncRNA MBNL1-AS1 in accordance with the instructions of Lipofectamine 2000 (Invitrogen, Carlsbad, CA, USA) (*p* < 0.05, Fig. 3e). CCK-8 showed that the cell viability of NSCLC CSCs decreased significantly after treatment with an overexpression of lncRNA MBNL1-AS1 (Fig. 3f), along with a significantly decreased 50% inhibition concentration (IC50) value of gefitinib and cisplatin in cells treated with oe-lncRNA MBNL1-AS1 (Fig. 3g), suggesting that lncRNA MBNL1-AS1 overexpression increased the sensitivity of A549 CSCs to anti-tumor drugs. Clone formation assay in vitro, scratch test, transwell assay, and sphere formation highlighted the evidently reduced proliferation, migration, invasion, and sphere formation of NSCLC CSCs in cells treated with oe-lncRNA MBNL1-AS1 (Fig. 3h–k). In addition, western blot analysis revealed that the protein levels of endogenous stem genes of NSCLC CSCs (Oct4 and ABCG2) in cells treated with oe-lncRNA MBNL1-AS1 were lower than the protein levels observed in cells treated with oe-NC (*p* < 0.05, Fig. 3l). These results suggested that restored lncRNA MBNL1-AS1 inhibited CSC proliferation, migration, invasion, drug resistance, and sphere formation in NSCLC.

### LncRNA MBNL1-AS1 negatively regulates miR-301b-3p

**Fig. 4:**
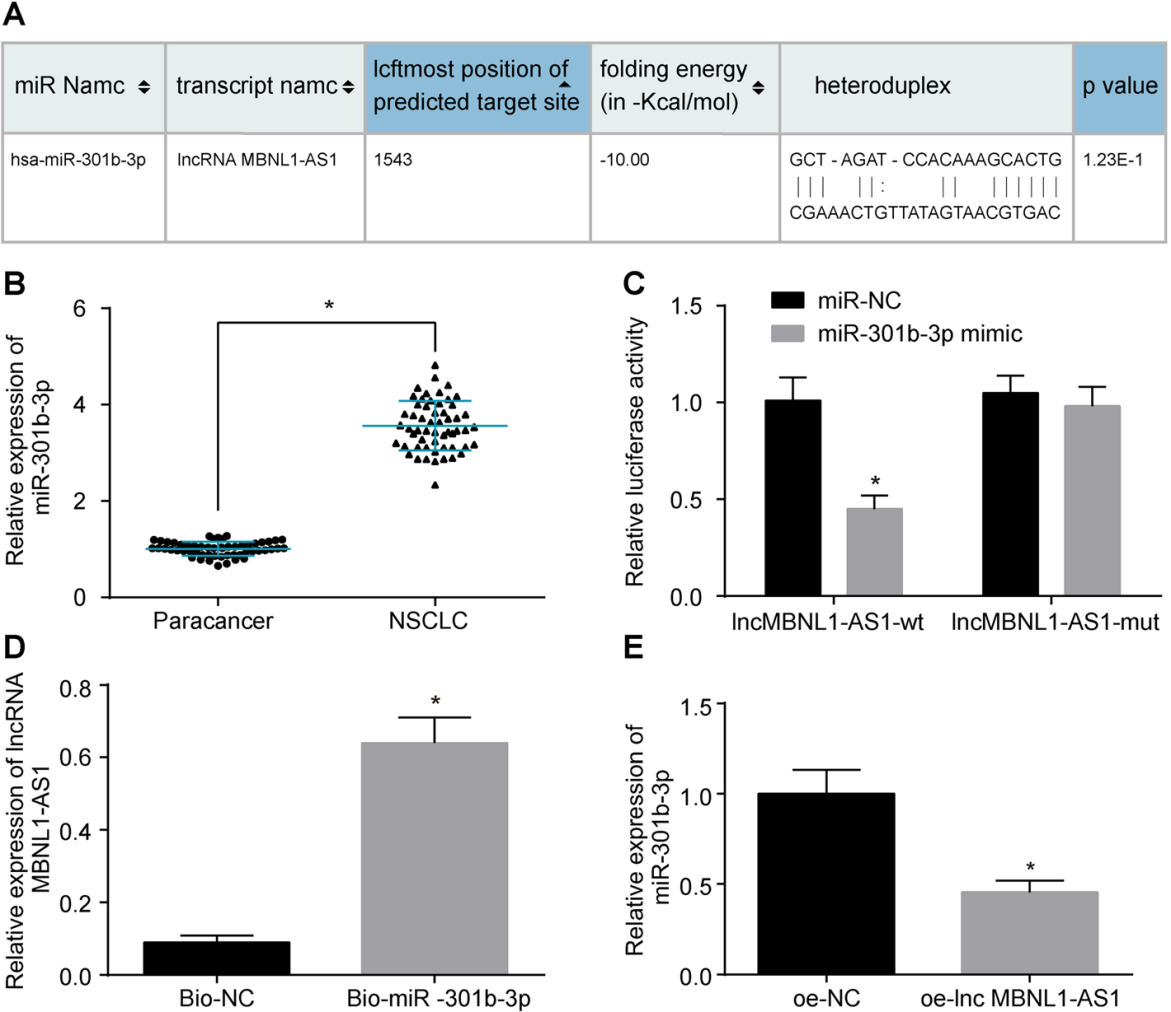
LncRNA MBNL1-AS1 negatively regulates miR-301b-3p. **a** Sequence complementarity between lncRNA MBNL1-AS1 and miR-301b-3p detected by bioinformatics analysis. **b** miR-301b-3p expression in NSCLC tissues and adjacent normal tissues measured using RT-qPCR (*n* = 56). **c** Luciferase activity. **d** Binding situation between lncRNA MBNL1-AS1 and miR-301b-3p verified using RNA pull-down. **e** miR-301b-3p expression in A549-SP^+^ after lncRNA MBNL1-AS1 overexpression measured using RT-qPCR; *p < 0.05 vs. the adjacent normal tissues, co-transfection with miR-NC, and lncRNA MBNL1-AS1-wt. Measurement data were depicted as mean ± standard deviation. **b** Data was analyzed using the paired *t* test. **c**–**e** Comparisons between two groups were analyzed using unpaired *t* test. The experiment was repeated three times

Succeeding results indicated that lncRNA MBNL1-AS1 elevation contributed to the alleviation of CSC proliferation, invasion, migration, drug resistance, and sphere formation in NSCLC, which led to the identification of the target relationship between lncRNA MBNL1-AS1 and miR-301b-3p. As shown in Fig. 4a, bioinformatics analysis verified the presence of sequence complementarity between lncRNA MBNL1-AS1 and miR-301b-3p. RT-qPCR was performed to measure the miR-301b-3p expression in 56 NSCLC tissues and adjacent normal tissues (Fig. 4b), which highlighted a higher miR-301b-3p expression in the NSCLC tissues than the adjacent normal tissues (*p* < 0.05). The results of dual-luciferase reporter gene assay demonstrated a reduced luciferase activity upon co-transfection with lncRNA MBNL1-AS1-wt and miR-301b-3p mimic in A549-SP^+^, suggesting that miR-301b-3p was specifically bound to lncRNA MBNL1-AS1 (*p* < 0.05, Fig. 4c). Moreover, the results of RNA pull-down indicated an evident increase in the content of lncRNA MBNL1-AS1 enriched by biotinylated miR-301b-3p (Fig. 4d). Meanwhile, the miR-301b-3p expression in A549-SP^+^ treated with oe-lncRNA MBNL1-AS1 had markedly reduced (Fig. 4e). The above results further prove that lncRNA MBNL1-AS1 could target miR-301b-3p.

### miR-301b-3p reverses the inhibitory effects of lncRNA MBNL1-AS1 on A549-SP^+^

**Fig. 5:**
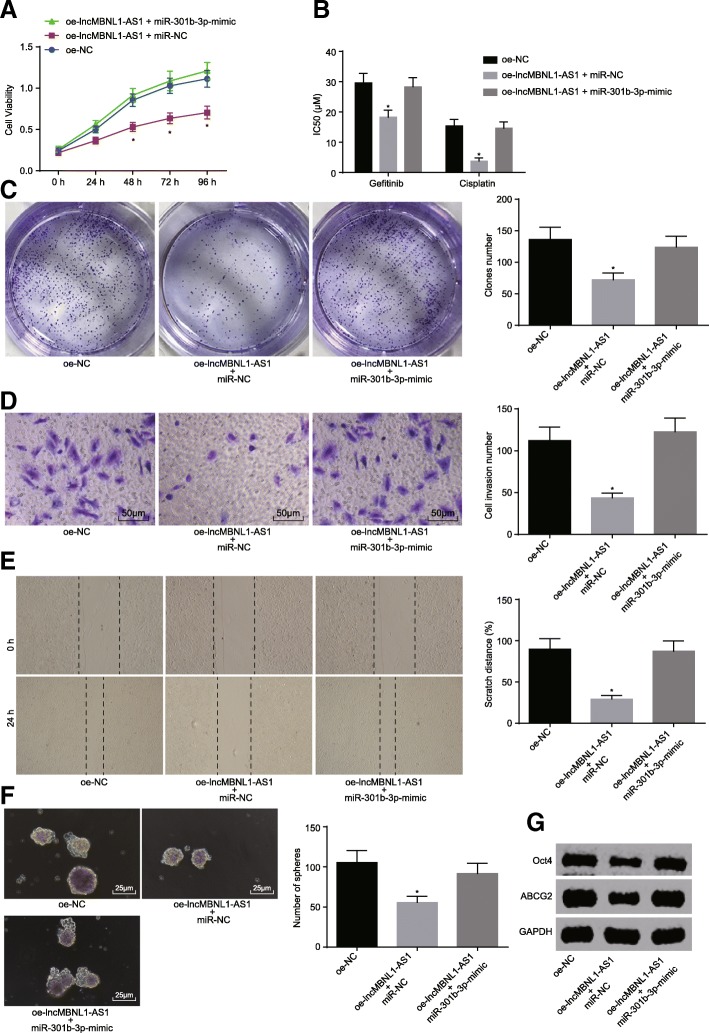
miR-301b-3p reverses the suppressive effects of lncRNA MBNL1-AS1 on A549-SP^+^. **a** Proliferation of A549-SP^+^ after lncRNA MBNL1-AS1 and miR-301b-3p overexpression detected using CCK-8. **b** Drug resistance of A549-SP^+^ against anti-tumor drugs after lncRNA MBNL1-AS1 and miR-301b-3p overexpression detected using CCK-8. **c** Clone formation of A549-SP^+^ detected after lncRNA MBNL1-AS1 and miR-301b-3p overexpression using clone formation assay. **d** Cell invasion of A549-SP^+^ after lncRNA MBNL1-AS1 and miR-301b-3p overexpression detected using transwell assay. **e** Cell migration of A549-SP^+^ after lncRNA MBNL1-AS1 and miR-301b-3p overexpression detected using scratch test. **f** Sphere formation of A549-SP^+^ after lncRNA MBNL1-AS1 and miR-301b-3p overexpression detected using sphere formation assay. **g** Protein levels of Oct4, ABCG2 in A549-SP^+^ after lncRNA MBNL1-AS1, and miR-301b-3p overexpression assessed using western blot analysis; *p < 0.05 vs. A549 cells treated with oe-NC. Measurement data were depicted as mean ± standard deviation. Comparisons among multiple groups analyzed using one-way ANOVA with Tukey post hoc test used. The experiment was repeated three times

With the aforementioned results highlighting the relationship between lncRNA MBNL1-AS1 and miR-301b-3p, the primary focus of the experiment was driven towards exploring the effect of miR-301b-3p on A549-SP^+^. Firstly, following the instructions of Lipofectamine 2000 (Invitrogen, Carlsbad, CA, USA), oe-lncRNA MBNL1-AS1 was transported into A549-SP^+^ with or without miR-301b-3p mimic. CCK-8, clone formation assay, scratch test, and transwell assay displayed that lncRNA MBNL1-AS1 elevation suppressed the cell viability, IC50 value, migration, and invasion of A549-SP^+^ (Fig. 5a–e), whereas miR-301b-3 rescued A549-SP^+^ from lncRNA MBNL1-AS1. Meanwhile, the sphere formation assay and western blot analysis implied that lncRNA MBNL1-AS1 restoration also reduced sphere formation and levels of Oct4 and ABCG2 of A549-SP^+^ (Fig. 5f and g), and miR-301b-3 inverted the effect of lncRNA MBNL1-AS1 on sphere formation and levels of Oct4 and ABCG2. The obtained data is supportive that miR-301b-3p could inverse the inhibitory effects of lncRNA MBNL1-AS1 on A549-SP^+^.

### LncRNA MBNL1-AS1 regulates TGFBR2 by competitively binding to miR-301b-3p

**Fig. 6:**
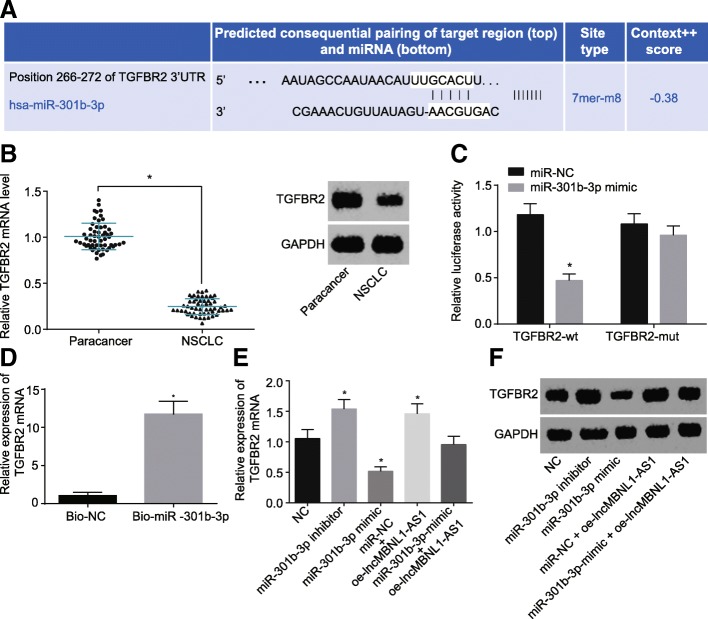
LncRNA MBNL1-AS1 increases TGFBR2 expression via competitive combination with miR-301b-3p. **a** Sequence complementarity between miR-301b-3p and TGFBR2 3′-UTR detected by bioinformatics analysis. **b** mRNA and protein levels of TGFBR2 in NSCLC tissues and adjacent normal tissues measured using RT-qPCR and western blot analysis (*n* = 56). **c** Luciferase activity after co-transfection. **d** Binding situation between miR-301b-3p and TGFBR2 verified using RNA pull-down. **e** TGFBR2 mRNA level in A549-SP^+^ measured using RT-qPCR. **f** TGFBR2 protein level in A549-SP^+^ measured using western blot analysis. NC group: cells treated with NC. miR-301b-3p-inhibitor group: cells treated with miR-301b-3p inhibition. miR-301b-3p-mimic group: cells treated with miR-301b-3p overexpression. miR-NC + oe-lncRNA MBNL1-AS1 group: cells treated with lncRNA MBNL1-AS1 overexpression and miR-NC. miR-301b-3p-mimic + oe-lncRNA MBNL1-AS1 group: cells treated with lncRNA MBNL1-AS1 overexpression and miR-301b-3p overexpression; *p < 0.05 vs. the adjacent normal tissues, co-transfection with miR-NC and TGFBR2-wt, A549 cells treated with Bio-NC, or A549 cells treated with NC. Measurement data were depicted as mean ± standard deviation. **b** Data was analyzed using the paired *t* test. **c**, **d** Comparisons between two groups were analyzed using unpaired *t* test. **e** Comparisons among multiple groups analyzed using one-way ANOVA with Tukey post hoc test used. The experiment was repeated three times. Bio, biotin

With successful data verification of the role of miR-301b-3p in executing the anti-tumor effect of lncRNA MBNL1-AS1 in A549-SP^+^, the focus of the experiment was to further study the mechanisms of miR-301b-3p in executing the anti-tumor effect of lncRNA MBNL1-AS1. RT-qPCR and western blot analysis were performed to determine the TGFBR2 mRNA and protein levels in 56 NSCLC tissues and adjacent normal tissues, which showed that the TGFBR2 mRNA and protein levels were lower in the NSCLC tissues than the adjacent normal tissues (*p* < 0.05, Fig. 6b). Moreover, the dual-luciferase reporter gene assay demonstrated with reduced luciferase activity in A549-SP^+^ co-transfected with miR-301b-3p-mimic and TGFBR2-wt (Fig. 6 a and c). Simultaneously, RNA pull-down showed an elevated mRNA level of TGFBR2 enriched in bio-miR-301b-3p (Fig. 6d), suggesting that miR-301b-3p could specifically bind to TGFBR2. In order to further verify whether lncRNA MBNL1-AS1 regulates the TGFBR2 expression by competitively binding to miR-301b-3p, the miR-301b-3p-inhibitor, miR-301b-3p-mimic, and oe-lncRNA MBNL1-AS1 plasmids were introduced into the cells following the instructions of Lipofectamine 2000 (Invitrogen, Carlsbad, CA, USA). RT-qPCR and western blot analysis presented that restored miR-301b-3p decreased the TGFBR2 expression, while miR-301b-3p depletion or lncRNA MBNL1-AS1 elevation increased the TGFBR2 expression in A549-SP^+^, and elevated lncRNA MBNL1-AS1 could internalize the effects of miR-301b-3p restoration on TGFBR2 expression (Fig. 6e and f). Altogether, the results demonstrated TGFBR2 as a direct target of miR-301b-3p, upregulation of which could inhibit the TGFBR2 expression. After inhibition of miR-301b-3p, lncRNA MBNL1-AS1 restoration could stimulate the TGFBR2 expression in A549-SP^+^. LncRNA MBNL1-AS1 annulled the suppressive effects of miR-301b-3p on TGFBR2 expression, indicating that lncRNA MBNL1-AS1 could indirectly upregulate TGFBR2 via a competitive combination with miR-301b-3p.

### LncRNA MBNL1-AS1 and miR-301b-3p regulate biological activity of A549-SP^+^ via TGF-β pathway

**Fig. 7:**
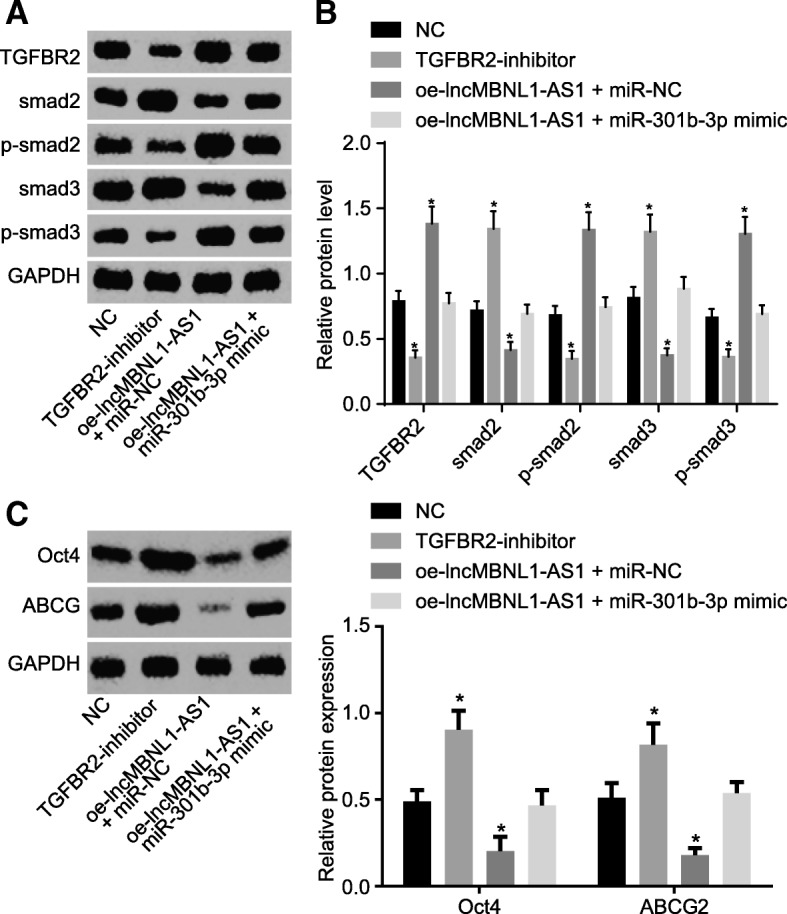
LncRNA MBNL1-AS1 and miR-301b-3p regulate the TGF-β pathway to modulate the biological activity of A549-SP^+^. **a**, **b** Levels of TGFBR2, smad2/3, and p-smad2/3 determined using western blot analysis. **c** Levels of Oct4 and ABCG2 in A549-SP^+^ assessed using immunofluorescence staining; *p < 0.05 vs. A549 cells treated with NC. Measurement data were depicted as mean ± standard deviation. Comparisons among multiple groups were analyzed using one-way ANOVA with Tukey post hoc test used. The experiment was repeated three times

After the establishment of results demonstrating that lncRNA MBNL1-AS1 regulates TGFBR2 by competitively binding to miR-301b-3p, the focus of the experiment then shifted to explore how a combination of lncRNA MBNL1-AS1 with miR-301b-3p exercises its effects on the TGF-β pathway, thereby regulating the biological activity in A549-SP^+^. TGFBR2 is regarded as a member of the TGF-β pathway [23]. Thus, we employed western blot analysis to investigate whether lncRNA MBNL1-AS1 and miR-301b-3p affect the biological activity of A549-SP^+^ by mediating the TGF-β pathway. The results displayed that inhibition of TGF-β reduced the levels of TGFBR2 and phosphorylated smad2/3, while the elevated levels of smad2/3, Oct4, and ABCG2, while overexpression of lncRNA MBNL1-AS1 + miR-301b-3p-NC exhibited an opposite trend (Fig. 7a–c). Furthermore, miR-301b-3p mimic reversed the changes of TGFBR2, phosphorylated smad2/3, smad2/3, Oct4, and ABCG2 induced by oe-lncRNA MBNL1-AS1. Altogether, lncRNA MBNL1-AS1 could facilitate the application of biological activity of the TGF-β pathway in A549-SP^+^, while the impact of miR-301b-3p on the biological activity of the TGF-β pathway in A549-SP^+^ was repressed.

### Restored lncRNA MBNL1-AS1 or depleted miR-301b-3p inhibits xenograft tumor formation in nude mice

**Fig. 8:**
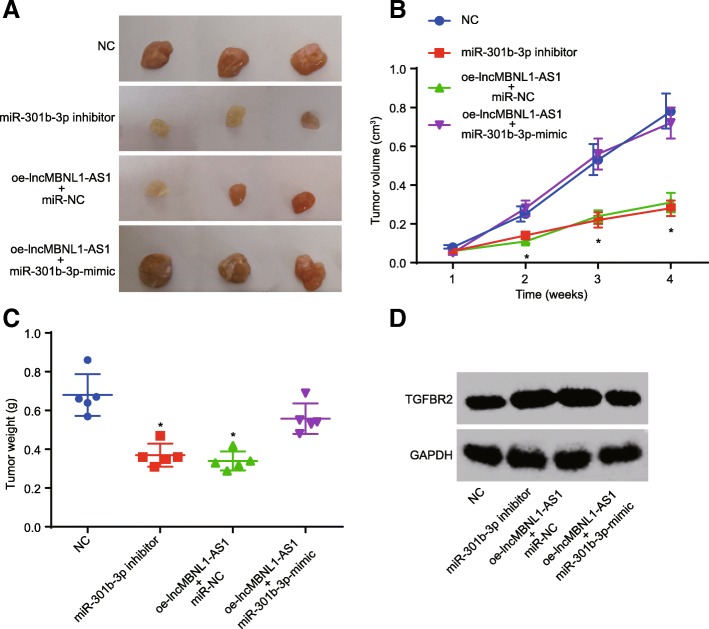
Overexpressed lncRNA MBNL1-AS1 or downregulated miR-301b-3p suppresses xenograft tumor formation in nude mice. **a** Xenograft tumor formation in nude mice. **b** Tumor volume of nude mice. **c** Tumor weight of nude mice. **d** TGFBR2 protein level determined using western blot analysis. NC group: mice treated with NC. miR-301b-3p-inhibitor group: mice treated with miR-301b-3p inhibition. miR-301b-3p-mimic group: mice treated with miR-301b-3p overexpression. miR-NC + oe-lncRNA MBNL1-AS1 group: mice treated with lncRNA MBNL1-AS1 overexpression and miR-NC. miR-301b-3p-mimic + oe-lncRNA MBNL1-AS1 group: mice treated with lncRNA MBNL1-AS1 overexpression and miR-301b-3p overexpression; *p < 0.05 vs. nude mice treated with NC. Measurement data were depicted as mean ± standard deviation. Comparisons among multiple groups were analyzed using one-way ANOVA with Tukey post hoc test used. The experiment was repeated three times

With findings supporting the regulation of the biological activity of A549-SP^+^ via the TGF-β pathway due to lncRNA MBNL1-AS1 and miR-301b-3p via the TGF-β pathway, our last objective was to evaluate whether the restored lncRNA MBNL1-AS1 or depleted miR-301b-3p affected the xenograft tumor formation in nude mice. This experiment was commenced by the transportation of oe-lncRNA MBNL1-AS1, miR-301b-3p-inhibitor, and oe-lncRNA MBNL1-AS1 + miR-301b-3p-mimic into A549-SP^+^ following the instructions of Lipofectamine 2000 (Invitrogen, Carlsbad, CA, USA). Next, the transfected A549-SP^+^ were subcutaneously injected into the armpits of female BALB/c nude mice, which indicated that miR-301b-3p depletion or lncRNA MBNL1-AS1 elevation reduced the mean tumor volume and weight, and upregulation of both lncRNA MBNL1-AS1 and miR-301b-3p contributed to normal tumor formation (Fig. 8a–c). Western blot analysis indicated that miR-301b-3p depletion or lncRNA MBNL1-AS1 restoration elevated the TGFBR2 protein level (Fig. 8d). With the aforementioned results serving as the theoretical basis, it was speculated that restored lncRNA MBNL1-AS1 or depleted miR-301b-3p inhibited the xenograft tumor formation in nude mice and increased the TGFBR2 protein level.

## Discussion

In recent years, CSCs with higher tumorigenicity have prevailed as an efficacious approach to treat NSCLC, while the understanding of the characteristics of CSCs in NSCLC therapies is still a withstanding topic of discussion [6, 8]. Accumulated evidences have indicated the involvement of lncRNAs in vital processes of NSCLC via functioning as mediators of CSCs [24]. The current study was performed with an aim to explain the suppressive role of lncRNA MBNL1-AS1 in NSCLC in an attempt to further elucidate the underlying mechanism associated with miR-301b-3p-targeted TGFBR2. The key observations of the current study were primarily centered around the protection of CSC cells from the degrading nature of proliferation, invasion, drug resistance, and sphere formation in NSCLC by activating TGFBR2 via a blockade of the miR-301b-3p upon restoration of lncRNA MBNL1-AS1 expression.

Initially, the current study revealed that lncRNA MBNL1-AS1 is downregulated in NSCLC tissues and cells. The dysregulation of lncRNA expression has been acknowledged as an implication in the tumorigenesis of NSCLC [9]. Upon detection of the expression of several lncRNAs, the lncRNA HMlincRNA717 and lncRNA taurine-upregulated gene 1 (TUG1) were clinically proven to be poorly expressed in NSCLC and associated with an inferior prognosis [25, 26]. Our findings were consistent with the findings of an existing research, which demonstrated the lncRNA MBNL1-AS1 to be poorly expressed in NSCLC [12]. Moreover, the current study disclosed the functionality of lncRNA MBNL1-AS1 as a molecular sponge of miR-301b-3p, due to its persistently high expression in NSCLC tissues and cells. A previous study demonstrated the liberating property of lncRNAs on various RNA transcripts by functioning as ceRNAs to implicitly regulate miRs, and lncRNAs participate in ceRNA networks and mRNA-miRNA-lncRNA crosstalk, which is implicated in human disease [27]. An existing research observed that lncRNA MBNL1 was bound with C allelic pre-miR-1307, which led to a reduced expression of miR-1307-3p, thus increasing the susceptibility to colorectal cancer [11]. An aberrant expression of miRNAs has been observed in the pathogenesis of several diseases, including various human cancers [28]. Shi et al. indicated an overexpression of miR-301a in NSCLC tissues [29]. Furthermore, a research observed an upregulated miR-301b expression in lung cancer tissues and cells [16]. The results of biological analysis and relevance analysis further verified TGFBR2 as a target gene of miR-301b-3p, which has a positive correlation with lncRNA MBNL1-AS1. A previous study speculated a consistently poor expression of TGFBR2 in NSCLC [30]. A study elucidated miR-301b to be a member of the pan-cancer oncogenic miRNA superfamily, which targets TGFBR2 [31]. The aforementioned evidences support the speculation that lncRNA MBNL1-AS1 is downregulated while miR-301b-3p is upregulated in NSCLC, with a positive correlation of lncRNA MBNL1-AS1 with TGFBR2 by functioning as a sponge of miR-301b-3p.

In addition, the data from this current study supports the speculation that upregulated lncRNA MBNL1-AS1 or inhibited miR-301b-3p suppresses CSC proliferation, invasion, migration, drug resistance, sphere formation, and tumor formation in NSCLC by upregulating TGFBR2. An existing research confirmed a stimulated proliferation in combination with suppressed apoptosis of the skeletal muscle cells upon lncRNA MBNL1-AS1 upregulation [32]. Liu et al. also proved that lncRNA maternally expressed gene 3 (MEG3) restoration led to highly stimulated drug sensitivity both in vitro and in vivo, with emphasis on the inhibitory impact of elevated MEG3 on drug resistance [33]. A research demonstrated miR-301b to stimulate cell invasion and facilitate drug resistance in pancreatic carcinoma [34]. Moreover, silencing of miR-301a could suppress cell proliferation, migration, and invasion in NSCLC, which implied to the functionality of miR-301a as a potential therapeutic strategy in NSCLC treatment [35]. Previous studies have proven that excised miR-301b could consequently inhibit bladder cancer cell proliferation, migration, and invasion [36]. Li et al. have elicited that miR-9-5p facilitated the proliferation and invasion of NSCLC cells by inhibiting the TGFBR2 expression [37], which compelled to the involvement of TGFBR2 silencing in the mechanism of miR-9-5p in regard to the proliferation and invasion of NSCLC cells. A previous study observed and credited the suppressive role of TGFBR2 in the carcinogenesis in NSCLC [17]. The abovementioned findings as the basis for this study suggest that lncRNA MBNL1-AS1 elevation suppresses the proliferation, invasion, drug resistance, and sphere formation of CSCs in NSCLC by inhibiting miR-301b-3p via upregulation of TGFBR2.

## Conclusions

**Fig. 9:**
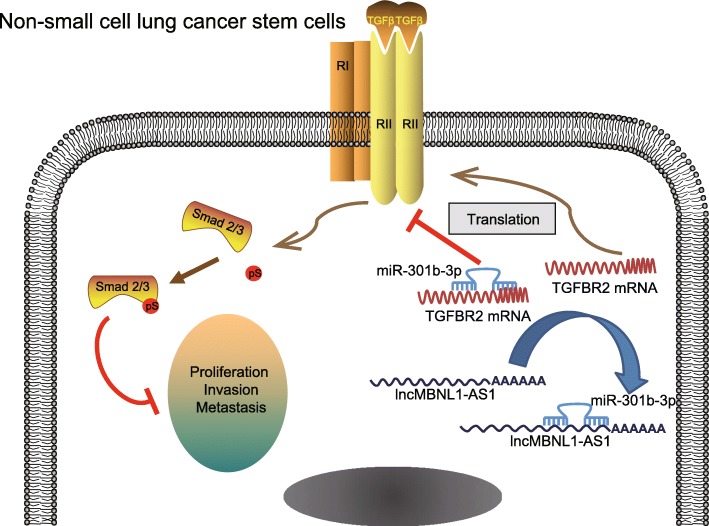
Potential mechanism of lncRNA MBNL1-AS1 involved in the biological activity of NSCLC CSCs via regulation of TGFBR2. In NSCLC CSCs, lncRNA MBNL1-AS1 sponged to miR-301b-3p that negatively regulated TGFBR2, and downregulation of lncRNA MBNL1-AS1 weakened the adsorption capacity, increased miR-301b-3p expression, and reduced TGFBR2 level, thus promoting the proliferation, migration, invasion, drug resistance, and sphere formation yet suppressing the activation of TGF-β pathway. By contrast, lncRNA MBNL1-AS1 overexpression strengthened the adsorption capacity of miR-301b-3p, accompanied by elevated TGFBR2 expression, hence resulting in a decline of the proliferation, migration, invasion, drug resistance, and sphere formation yet an increase in activation of TGF-β pathway

To conclude, the current study serves as evidence supporting that lncRNA MBNL1-AS1 restoration could decelerate the occurrence and progression of NSCLC, thereby highlighting the functionality of lncRNA MBNL1-AS1 restoration as a sponge of miR-301b-3p to suppress the proliferation, invasion, drug resistance, and sphere formation of CSC cells in NSCLC via upregulation of TGFBR2 (Fig. 9). Thus, the lncRNA MBNL1-AS1-miR-301b-3p network could facilitate as a novel aspect in NSCLC treatment. This study may serve as a potential insight for developing efficacious therapeutic treatment strategies for NSCLC treatment.

## Abbreviations

3′-UTR: 3′-Untranslated region
ABCG2: ATP-binding cassette superfamily G (White) member 2
ANOVA: Analysis of variance
ATCC: American Type Culture Collection
CCK-8: Cell counting kit-8
CRC: Colorectal cancer
CSCs: Cancer stem cells
DAPI: 4′6-Diamidino-2-phenylindole
DEGs: Differentially expressed genes
DMEM: Dulbecco’s modified Eagle medium
FBS: Fetal bovine serum
FISH: Fluorescence in situ hybridization
GAPDH: Glyceraldehyde-3-phosphate dehydrogenase
GEO: Gene Expression Omnibus
IgG: Immunoglobulin G
lncRNAs: Long non-coding RNAs
miRNAs/miRs: MicroRNAs
mut: Mutant
NC: Negative control
NSCLC: Non-small cell lung cancer
OD: Optical density
PBS: Phosphate-buffered saline
PMSF: Phenylmethyl sulfonylfluoride
PVDF: Polyvinylidene fluoride
RIPA: Radio-immunoprecipitation assay
SD: Standard deviation
SDS-PAGE: Sodium dodecyl sulfate-polyacrylamide gel electrophoresis
SMAD: Sekclsky mothers against dpp
Wt: Wild type

## References

[1] Junttila MR, Karnezis AN, Garcia D (2010). Selective activation of p53-mediated tumour suppression in high-grade tumours. Nature..

[2] Ceppi P, Mudduluru G, Kumarswamy R (2010). Loss of miR-200c expression induces an aggressive, invasive, and chemoresistant phenotype in non-small cell lung cancer. Mol Cancer Res.

[3] Foss KM, Sima C, Ugolini D (2011). miR-1254 and miR-574-5p: serum-based microRNA biomarkers for early-stage non-small cell lung cancer. J Thorac Oncol.

[4] Fehrenbacher L, Spira A, Ballinger M (2016). Atezolizumab versus docetaxel for patients with previously treated non-small-cell lung cancer (POPLAR): a multicentre, open-label, phase 2 randomised controlled trial. Lancet..

[5] Perona R, Lopez-Ayllon BD, de Castro Carpeno J (2011). A role for cancer stem cells in drug resistance and metastasis in non-small-cell lung cancer. Clin Transl Oncol.

[6] Zhao C, Setrerrahmane S, Xu H (2015). Enrichment and characterization of cancer stem cells from a human non-small cell lung cancer cell line. Oncol Rep.

[7] Holmboe S, Hansen PL, Thisgaard H (2017). Evaluation of somatostatin and nucleolin receptors for therapeutic delivery in non-small cell lung cancer stem cells applying the somatostatin-analog DOTATATE and the nucleolin-targeting aptamer AS1411. PLoS One.

[8] Zakaria N, Satar NA, Abu Halim NH (2017). Targeting lung cancer stem cells: research and clinical impacts. Front Oncol.

[9] Nie FQ, Sun M, Yang JS (2015). Long noncoding RNA ANRIL promotes non-small cell lung cancer cell proliferation and inhibits apoptosis by silencing KLF2 and P21 expression. Mol Cancer Ther.

[10] Fu Y, Ramisetty SR, Hussain N (2012). MBNL1-RNA recognition: contributions of MBNL1 sequence and RNA conformation. Chembiochem..

[11] Tang R, Qi Q, Wu R (2015). The polymorphic terminal-loop of pre-miR-1307 binding with MBNL1 contributes to colorectal carcinogenesis via interference with Dicer1 recruitment. Carcinogenesis..

[12] Yu H, Xu Q, Liu F (2015). Identification and validation of long noncoding RNA biomarkers in human non-small-cell lung carcinomas. J Thorac Oncol.

[13] Mo X, Zhang F, Liang H (2014). miR-544a promotes the invasion of lung cancer cells by targeting cadherina 1 in vitro. Onco Targets Ther.

[14] Bertero T, Cottrill K, Krauszman A (2015). The microRNA-130/301 family controls vasoconstriction in pulmonary hypertension. J Biol Chem.

[15] Xue J, Xue J, Zhang J (2017). miR-130b-3p/301b-3p negatively regulated Rb1cc1 expression on myogenic differentiation of chicken primary myoblasts. Biotechnol Lett.

[16] Wu D, Chen B, Cui F (2016). Hypoxia-induced microRNA-301b regulates apoptosis by targeting Bim in lung cancer. Cell Prolif.

[17] Ma ZL, Hou PP, Li YL (2015). MicroRNA-34a inhibits the proliferation and promotes the apoptosis of non-small cell lung cancer H1299 cell line by targeting TGFbetaR2. Tumour Biol.

[18] Xu JB, Bao Y, Liu X (2007). Defective expression of transforming growth factor beta type II receptor (TGFBR2) in the large cell variant of non-small cell lung carcinoma. Lung Cancer.

[19] Puri KS, Suresh KR, Gogtay NJ (2009). Declaration of Helsinki, 2008: implications for stakeholders in research. J Postgrad Med.

[20] Yu CC, Hu FW, Yu CH (2016). Targeting CD133 in the enhancement of chemosensitivity in oral squamous cell carcinoma-derived side population cancer stem cells. Head Neck.

[21] Cao S, Wang Z, Gao X (2018). FOXC1 induces cancer stem cell-like properties through upregulation of beta-catenin in NSCLC. J Exp Clin Cancer Res.

[22] Rojo AI, Medina-Campos ON, Rada P (2012). Signaling pathways activated by the phytochemical nordihydroguaiaretic acid contribute to a Keap1-independent regulation of Nrf2 stability: role of glycogen synthase kinase-3. Free Radic Biol Med.

[23] Yang H, Zhang H, Zhong Y (2017). Concomitant underexpression of TGFBR2 and overexpression of hTERT are associated with poor prognosis in cervical cancer. Sci Rep.

[24] Wang R, Dong HX, Zeng J (2018). LncRNA DGCR5 contributes to CSC-like properties via modulating miR-330-5p/CD44 in NSCLC. J Cell Physiol.

[25] Xie X, Liu HT, Mei J (2014). LncRNA HMlincRNA717 is down-regulated in non-small cell lung cancer and associated with poor prognosis. Int J Clin Exp Pathol.

[26] Lin PC, Huang HD, Chang CC (2016). Long noncoding RNA TUG1 is downregulated in non-small cell lung cancer and can regulate CELF1 on binding to PRC2. BMC Cancer.

[27] Lv J, Fan HX, Zhao XP (2016). Long non-coding RNA Unigene56159 promotes epithelial-mesenchymal transition by acting as a ceRNA of miR-140-5p in hepatocellular carcinoma cells. Cancer Lett.

[28] Zhang JG, Wang JJ, Zhao F (2010). MicroRNA-21 (miR-21) represses tumor suppressor PTEN and promotes growth and invasion in non-small cell lung cancer (NSCLC). Clin Chim Acta.

[29] Shi YK, Zang QL, Li GX (2016). Increased expression of microRNA-301a in nonsmall-cell lung cancer and its clinical significance. J Cancer Res Ther.

[30] Zhang HT, Chen XF, Wang MH (2004). Defective expression of transforming growth factor beta receptor type II is associated with CpG methylated promoter in primary non-small cell lung cancer. Clin Cancer Res.

[31] Fort RS, Matho C, Oliveira-Rizzo C (2018). An integrated view of the role of miR-130b/301b miRNA cluster in prostate cancer. Exp Hematol Oncol.

[32] Li XF, Wang ZQ, Li LY (2018). Downregulation of the long noncoding RNA MBNL1-AS1 protects sevoflurane-pretreated mice against ischemia-reperfusion injury by targeting KCNMA1. Exp Mol Med.

[33] Wang L, Ma L, Xu F (2018). Role of long non-coding RNA in drug resistance in non-small cell lung cancer. Thorac Cancer.

[34] Funamizu N, Lacy CR, Parpart ST (2014). MicroRNA-301b promotes cell invasiveness through targeting TP63 in pancreatic carcinoma cells. Int J Oncol.

[35] Wu Z, Li Y, Zhang G (2017). Downregulation of microRNA-301a inhibited proliferation, migration and invasion of non-small cell lung cancer by directly targeting DLC1. Oncol Lett.

[36] Yan L, Wang Y, Liang J (2017). MiR-301b promotes the proliferation, mobility, and epithelial-to-mesenchymal transition of bladder cancer cells by targeting EGR1. Biochem Cell Biol.

[37] Li G, Wu F, Yang H (2017). MiR-9-5p promotes cell growth and metastasis in non-small cell lung cancer through the repression of TGFBR2. Biomed Pharmacother.

